# Lipid accumulation product and endometriosis in women aged 18 years and older: A cross-sectional study of NHANES 1999 to 2006

**DOI:** 10.1097/MD.0000000000049437

**Published:** 2026-06-26

**Authors:** Xin Liu, Qingbao Pang, Boyu Sun, Jing Lv

**Affiliations:** a Harbin Youhao Jikun Traditional Chinese Medicine Clinic, Harbin, Heilongjiang, China; b Department of Ophthalmology, The First Affiliated Hospital of Heilongjiang University of Chinese Medicine, Harbin, Heilongjiang, China; c Department of General Surgery, The Second Affiliated Hospital of Heilongjiang University of Chinese Medicine, Harbin, Heilongjiang, China; d Department of Rehabilitation Medicine, Jinan Beicheng Hospital, Jinan, Shandong, China.

**Keywords:** dose–response relationship, endometriosis, epidemiology, lipid accumulation product, metabolic syndrome

## Abstract

Endometriosis, a chronic gynecological disorder, is increasingly linked to metabolic dysregulation. The lipid accumulation product (LAP) – a biomarker integrating waist circumference and triglycerides – may provide additional information on this association but remains insufficiently studied. We aimed to assess the association between LAP and endometriosis in a nationally representative cohort. Analyzing 1999 to 2006 National Health and Nutrition Examination Survey data from 1792 women with self-reported endometriosis status, we calculated LAP as (waist circumference [cm] − 58) × (triglycerides [mmol/L]). Multivariable logistic regression evaluated linear associations, while restricted cubic splines and threshold analyses assessed nonlinearity. Subgroup interactions were tested via stratified models. Women in the highest LAP quartile exhibited 70% greater endometriosis odds than the lowest quartile (odds ratio = 1.70, 95% confidence interval = 1.03–2.81, *P* < .05). Restricted cubic spline analyses supported an approximately linear dose–response pattern across the LAP distribution, with higher LAP values associated with progressively greater odds of endometriosis (*P* < .001). While most subgroups showed consistent associations, exploratory subgroup analyses suggested that the LAP-endometriosis association was stronger among women with a history of stroke (*P* < .05). Higher LAP values were associated with higher odds of endometriosis in this cross-sectional sample, with odds increasing progressively across the LAP distribution. LAP may be considered a potentially useful marker for metabolic risk stratification in women with possible endometriosis, but its clinical utility requires confirmation in prospective studies.

## 1. Introduction

Endometriosis, a prevalent gynecological disorder, is characterized by the ectopic implantation of endometrial tissue outside the uterine cavity.^[1,2]^ Its clinical manifestations – including severe dysmenorrhea, chronic pelvic pain, and infertility – significantly impair patients’ quality of life and reproductive capacity.^[3]^ Globally, the prevalence of endometriosis has risen steadily, affecting approximately 10% of women of reproductive age, with rates soaring to 30% to 50% among individuals with infertility. This condition profoundly impacts both physical and mental health, underscoring the paramount importance of early diagnosis and timely intervention to alleviate symptoms and preserve fertility. Current diagnostic approaches rely heavily on clinical symptoms, imaging modalities, and invasive surgical confirmation. However, limitations in these methods often delay detection, allowing disease progression.^[4]^ Recent advancements have spurred interest in identifying accessible biomarkers to facilitate earlier diagnosis.

The lipid accumulation product (LAP), a composite visceral adiposity-related metabolic index integrating waist circumference (WC) and triglyceride (TG) levels, provides a comprehensive assessment of metabolic dysregulation.^[5]^ Since its introduction, the LAP has outperformed body mass index (BMI) as a surrogate of visceral adiposity in several large cohorts. An early US study showed superior prediction of global metabolic risk, and 2 later population analyses confirmed better discrimination for both cardiovascular profile and diabetes identification.^[6–8]^ In reproductive-age women, a Brazilian cohort further validated LAP as a cardiometabolic marker in polycystic-ovary-syndrome patients.^[9]^ Evidence also links high LAP to incident metabolic syndrome and selected cancers, underscoring the formula’s robustness across clinical contexts.^[10,11]^ Because its 2 components – waist circumference and triglycerides – are hallmarks of abdominal obesity, a metabolic phenotype that has been linked to gynecologic morbidity and to endometriosis in some but not all epidemiologic studies,^[12,13]^ evaluating LAP in this gynecologic disorder is biologically plausible. Although findings on overall BMI and endometriosis are heterogeneous, central adiposity and related metabolic disturbances appear to be more consistently implicated.^[14]^ Mechanistically, severe obesity may exacerbate disease progression by altering hormonal homeostasis (e.g., estrogen dominance) and amplifying systemic inflammation.^[15,16]^ Elevated TG levels, serving as both metabolic and inflammatory markers, further potentiate these effects by disrupting lipid metabolism and fostering a pro-inflammatory microenvironment conducive to ectopic endometrial growth.^[17]^

This study investigates the association between LAP – a promising emerging biomarker – and endometriosis using data from the 1999 to 2006 National Health and Nutrition Examination Survey (NHANES). Through cross-sectional analysis with adjustment for major confounders, we aimed to further characterize the association between LAP and endometriosis in a nationally representative sample. Our findings may add complementary evidence for metabolic risk assessment and support future research on clinically accessible markers in women with suspected endometriosis.

## 2. Methods

The US NHANES, administered by the National Center for Health Statistics, serves as a population-level surveillance system monitoring health and nutritional metrics across demographic strata. Following ethical clearance from the National Center for Health Statistics Institutional Review Board, participants provided documented consent before enrollment. Because this study used publicly available, de-identified NHANES data, it was considered a secondary analysis of anonymized data and did not require additional institutional review board approval or new informed consent from participants. For this investigation, we extracted cross-sectional data spanning 4 consecutive surveys (1999–2006) to examine correlations between LAP and endometriosis prevalence. Comprehensive methodological documentation, including sampling protocols and variable definitions, is available publicly via the NHANES repository (https://www.cdc.gov/nchs/nhanes/).

### 2.1. Study population and design

**Figure 1.**
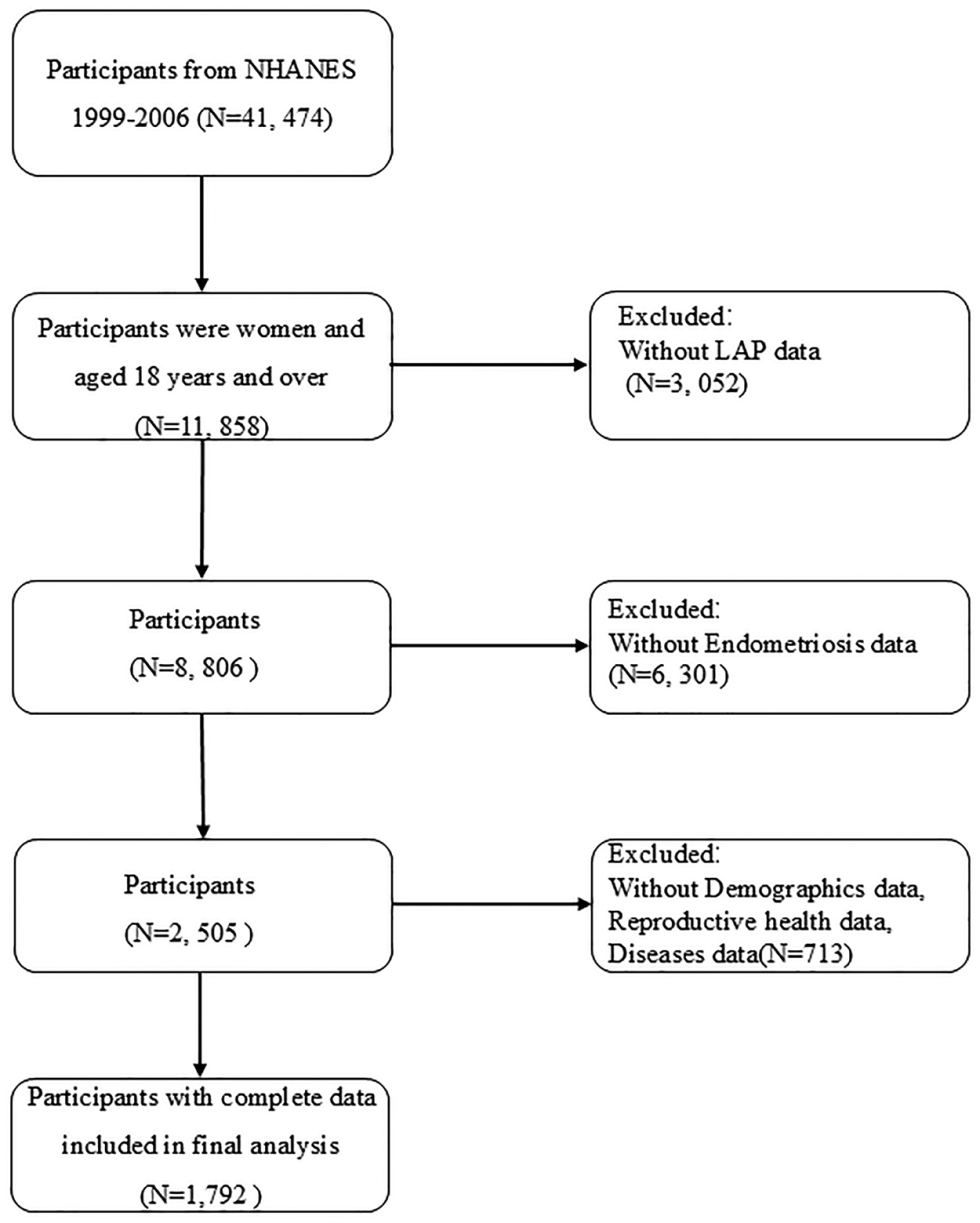
Flowchart of participant screening and enrollment. LAP = lipid accumulation product, NHANES = National Health and Nutrition Examination Survey.

The study initially included 41,474 participants from the 1999 to 2006 NHANES datasets. Eligible individuals were required to have complete demographic records, standardized anthropometric measurements, lipid profiles, hematological indices, and documented medical histories. These variables were considered as candidate covariates for baseline characterization, subgroup exploration, and confounding assessment. The final multivariable model retained the prespecified major confounders most directly related to demographic, socioeconomic, behavioral, and cardiometabolic status to preserve model parsimony and reduce the risk of overadjustment. Exclusion criteria encompassed individuals below 18 years of age, male participants, those with incomplete endometriosis diagnostic data or missing variables necessary for LAP calculation, and subjects lacking covariate data such as marital status, poverty-to-income ratio (PIR), contraceptive use, pregnancy history, alcohol consumption, smoking status, BMI, diabetes, asthma, arthritis, angina, congestive heart failure, coronary heart disease, liver disease, stroke, malignancy, or hypertension (Fig. 1).

### 2.2. Diagnosis of endometriosis

Endometriosis diagnostic data were obtained from self-administered health surveys completed by female NHANES participants across the 1999 to 2006. A standardized screening question (“Has a doctor or other health professional ever told you that you had endometriosis?”) was used to establish case status, with affirmative responses generating a dichotomous variable (present vs absent). Although the absence of imaging or surgical confirmation (e.g., laparoscopy or ultrasound) introduces potential recall bias inherent to self-reported diagnoses, we applied rigorous inclusion criteria by exclusively classifying participants reporting clinician-confirmed diagnoses as endometriosis cases to minimize misclassification errors.

### 2.3. LAP and assessment

Anthropometric evaluations and fasting blood sampling in NHANES were performed by certified examiners at designated field clinics following standardized protocols. WC measurements were obtained at the iliac crest midpoint using calibrated tape measures and recorded to the nearest centimeter. The LAP was calculated through the mathematical formula: (WC [cm] − 58) × fasting TG concentration (mmol/L). This sex-specific index was originally proposed in prior population research as a simple proxy of lipid overaccumulation by jointly capturing central adiposity and circulating TG, and it has since been widely applied and validated across epidemiologic cohorts. Although NHANES provides a complete lipid profile, we used TG in the LAP computation because TG is the lipid component embedded in the original, commonly adopted definition; retaining this established formulation also facilitates comparability with previous studies using LAP. Study subjects were subsequently stratified into quartiles (*Q*1–*Q*4) based on LAP values, with the lowest quartile (*Q*1) serving as the comparative baseline. This standardized operational protocol enhanced measurement precision while maintaining dataset integrity, facilitating robust statistical modeling in subsequent analytical phases.

### 2.4. Other covariates

To isolate the independent relationship between LAP and endometriosis, we systematically adjusted for confounding variables across 2 domains: sociodemographic/behavioral factors and clinical biomarkers.

Demographic parameters included age, self-reported race/ethnicity (Mexican American, Other Hispanic, non-Hispanic White, non-Hispanic Black, Other), and educational attainment, stratified as: <9th grade; 9th to 11th grade; high school diploma/general educational development; some college/associate degree; college graduate. Behavioral factors encompassed smoking history (coded no for < 100 lifetime cigarettes, yes for ≥ 100) and alcohol use (coded no for < 1 drink in the past 12 months, yes for ≥ 1), marital status (married/unmarried), and contraceptive practices.^[18]^ Socioeconomic status was quantified through the poverty-income ratio (PIR). Reproductive history variables included parity, menarche age, and pregnancy frequency. Comorbidities affecting metabolic profiles – asthma, arthritis, cardiovascular diseases (congestive heart failure, coronary artery disease, angina), cerebrovascular events, hepatic disorders, and malignancies – were recorded via physician-diagnosed yes/no responses. Anthropometric indices (height, WC, BMI [weight(kg)/height(m^2^)]), systolic/diastolic blood pressure (mean of triplicate measurements), and fasting serum lipids were analyzed. Hypertension was defined according to the Eighth Joint National Committee criteria (systolic blood pressure ≥ 140 mm Hg or diastolic blood pressure ≥ 90 mm Hg).^[19]^ Lipid panels evaluated TG, total cholesterol, low-density lipoprotein cholesterol, and high-density lipoprotein cholesterol.

### 2.5. Statistical analysis

All statistical procedures accounted for NHANES’s complex sampling design through the incorporation of survey weights, stratification variables, and clustered sampling units, adhering to the analytic guidelines of the Centers for Disease Control and Prevention. The weighting methodology addressed oversampling of minority populations, nonresponse adjustments, and post-stratification alignment with US Census demographic distributions. Categorical data were summarized as weighted proportions (%) and continuous variables as mean ± standard error. Between-group comparisons employed weighted Rao-Scott χ^2^ tests for categorical measures and Student *t* tests or Wald *F* tests for continuous parameters.

A tiered multivariable logistic regression framework evaluated LAP-endometriosis associations: model 1 (crude), model 2 (age, race, education-adjusted), and model 3 (adjustment for age, race, education, marital status, PIR, alcohol consumption, smoking status, physician-diagnosed diabetes, and hypertension). Nonlinear relationships were explored via restricted cubic splines (RCSs) with knot optimization, supplemented by threshold effect modeling to identify inflection points. Stratified analyses assessed subgroup heterogeneity through multiplicative interaction terms. All statistical analyses were performed using R software version 4.2.3 (R Foundation for Statistical Computing) with the survey package, and EmpowerStats version 2.0 (X&Y Solutions, Inc.).

## 3. Results

### 3.1. Characteristics of included subjects

The final analytical cohort included 1792 eligible women (from 41,474 NHANES 1999–2006 participants) with complete LAP measurements and self-reported, clinician-diagnosed endometriosis status. Stratified comparisons between endometriosis cases (n = 152) and controls (n = 1640) revealed significant intergroup disparities: affected individuals were older (39.9 ± 8.5 vs 35.7 ± 9.8 years, *P* < .001), more frequently identified as non-Hispanic White (71.05% vs 50.73%, *P* < .001), and exhibited higher educational attainment (college graduates: 26.32% vs 24.21%, *P* = .006). Metabolic profiles diverged substantially, with elevated TG levels (1.74 vs 1.46 mmol/L, *P* = .004) and increased contraceptive utilization (90.79% vs 81.10%, *P* = .003) in the endometriosis group. Lifestyle analysis demonstrated higher smoking prevalence (56.58% vs 43.17%, *P* = .001) and alcohol consumption rates (12.50% vs 7.87%, *P* = .047). Comorbidity burdens were markedly elevated in cases, including hypertension (24.34% vs 17.56%, *P* = .038), asthma (30.26% vs 14.57%, *P* < .001), arthritis (28.29% vs 13.17%, *P* < .001), stroke (4.61% vs 1.10%, *P* < .001), liver disease (4.61% vs 1.65%, *P* = .011), and malignancy (11.18% vs 4.33%, *P* < .001; Table 1).

**Table 1:** Weighted comparison in basic characteristics.

Characteristics	Overall	Endometriosis		*P* value
		Without	With	
	n = 1792	n = 1640	n = 152	
Age, y	36.100 ± 9.765	35.748 ± 9.804	39.901 ± 8.480	<.001
Height, cm	163.013 ± 6.649	162.988 ± 6.730	163.281 ± 5.718	.603
Waist circumference, cm	94.190 ± 15.931	94.064 ± 15.918	95.549 ± 16.066	.272
Age at first menstruation, y	12.558 ± 1.676	12.576 ± 1.666	12.362 ± 1.770	.131
HDL (mmol/L)	1.517 ± 0.423	1.518 ± 0.422	1.508 ± 0.435	.781
TC (mmol/L)	5.170 ± 1.157	5.157 ± 1.159	5.314 ± 1.136	.110
TG (mmol/L)	1.480 ± 1.170	1.456 ± 1.042	1.741 ± 2.090	.004
LDL (mmol/L)	2.982 ± 0.922	2.974 ± 0.922	3.069 ± 0.924	.235
Race (%)				<.001
Mexican American	357 (19.922%)	348 (21.220%)	9 (5.921%)	
Other Hispanic	80 (4.464%)	76 (4.634%)	4 (2.632%)	
Non-Hispanic White	940 (52.455%)	832 (50.732%)	108 (71.053%)	
Non-Hispanic Black	341 (19.029%)	315 (19.207%)	26 (17.105%)	
Other Race	74 (4.129%)	69 (4.207%)	5 (3.289%)	
Education level (%)				.006
<9th	109 (6.083%)	108 (6.585%)	1 (0.658%)	
9–11th	249 (13.895%)	236 (14.390%)	13 (8.553%)	
High school	386 (21.540%)	345 (21.037%)	41 (26.974%)	
Some college	611 (34.096%)	554 (33.780%)	57 (37.500%)	
College graduate	437 (24.386%)	397 (24.207%)	40 (26.316%)	
Marital status (%)				.216
Married	769 (42.913%)	711 (43.354%)	58 (38.158%)	
Unmarried	1023 (57.087%)	929 (56.646%)	94 (61.842%)	
PIR (%)				.187
<1	300 (16.741%)	279 (17.012%)	21 (13.816%)	
≥, <3	651 (36.328%)	602 (36.707%)	49 (32.237%)	
≥3	841 (46.931%)	759 (46.280%)	82 (53.947%)	
Drinking (%)				.047
Yes	148 (8.259%)	129 (7.866%)	19 (12.500%)	
No	1644 (91.741%)	1511 (92.134%)	133 (87.500%)	
BMI, kg/m^2^				.645
<25	639 (35.658%)	588 (35.854%)	51 (33.553%)	
≥25, <30	508 (28.348%)	467 (28.476%)	41 (26.974%)	
≥30	645 (35.993%)	585 (35.671%)	60 (39.474%)	
Smoking (%)				.001
Yes	794 (44.308%)	708 (43.171%)	86 (56.579%)	
No	998 (55.692%)	932 (56.829%)	66 (43.421%)	
Asthma (%)				<.001
Yes	285 (15.904%)	239 (14.573%)	46 (30.263%)	
No	1507 (84.096%)	1424 (86.829%)	106 (69.737%)	
Arthritis (%)				<.001
Yes	259 (14.453%)	216 (13.171%)	43 (28.289%)	
No	1533 (85.547%)	1424 (86.83%)	109 (71.711%)	
Congestive heart failure (%)				.004
Yes	13 (0.725%)	9 (0.549%)	4 (2.632%)	
No	1779 (99.275%)	1631 (99.451%)	148 (97.368%)	
Coronary heart disease (%)				.683
Yes	8 (0.446%)	7 (0.427%)	1 (0.658%)	
No	1784 (99.554%)	1633 (99.573%)	151 (99.342%)	
Angina (%)				.190
Yes	10 (0.558%)	8 (0.488%)	2 (1.316%)	
No	1782 (99.442%)	1632 (99.512%)	150 (98.684%)	
Stroke (%)				<.001
Yes	25 (1.395%)	18 (1.098%)	7 (4.605%)	
No	1767 (98.605%)	1632 (99.512%)	150 (98.684%)	
Liver disease (%)				.011
Yes	34 (1.897%)	27 (1.646%)	7 (4.605%)	
No	1758 (98.103%)	1613 (98.354%)	145 (95.395%)	
Malignancy (%)				<.001
Yes	88 (4.911%)	71 (4.329%)	17 (11.184%)	
No	1704 (95.089%)	1569 (95.671%)	135 (88.816%)	
Contraceptive use (%)				.003
Yes	1468 (81.920%)	1330 (81.098%)	138 (90.789%)	
No	324 (18.080%)	310 (18.902%)	14 (9.211%)	
Hypertension (%)				.038
Yes	325 (18.136%)	288 (17.561%)	37 (24.342%)	
No	1467 (81.864%)	1352 (82.439%)	115 (75.658%)	
Diabetes (%)				.657
Yes	71 (3.962%)	65 (3.963%)	6 (3.947%)	
No	1721 (96.038%)	1575 (96.037%)	146 (96.043%)	
LAP (%)				.320
≤21.460	448 (25.000%)	416 (25.366%)	32 (21.053%)	
21.470–58.880	448 (25.000%)	412 (25.122%)	36 (23.684%)	
58.890–76.520	448 (25.000%)	411 (25.061%)	37 (24.342%)	
>76.520	448 (25.000%)	401 (24.451%)	47 (30.921%)	

BMI = body mass index, HDL = high-density lipoprotein cholesterol, LAP = lipid accumulation product, LDL = low-density lipoprotein cholesterol, PIR = poverty-income ratio, TC = total cholesterol, TG = triglycerides.

### 3.2. Relationship between LAP and endometriosis

Survey-weighted logistic regression demonstrated a dose-dependent association between higher LAP and endometriosis odds across models (Table 2). In the crude model 1, LAP was positively associated with endometriosis when modeled as a continuous variable. After adjustment for age, race, and education in model 2, the association remained. In the fully adjusted model 3, additionally controlling for marital status, PIR, smoking, drinking, diabetes, and hypertension, women in the highest LAP quartile (Q4) had approximately 70% higher odds of endometriosis compared with those in the lowest quartile (Q1) (OR = 1.71, 95% confidence interval [CI] = 1.04–2.82; *P* < .05). Quartile-based sensitivity analysis showed a graded increase in odds across LAP quartiles. These findings collectively support an independent association between higher LAP and endometriosis across the applied modeling frameworks.

**Table 2:** Association between LAP and endometriosis.

	Continuous	*Q*1	*Q*2	*Q*3	*Q*4
Model 1 OR (95% CI)	1.002 (1.000–1.005)*	1	1.136 (0.692–1.864)	1.170 (0.715–1.915)	1.524 (0.953–2.437)
Model 2 OR (95% CI)	1.003 (1.001–1.005)*	1	1.177 (0.709–1.955)	1.243 (0.750–2.060)	1.745 (1.074–2.834)*
Model 3 OR (95% CI)	1.003 (1.000–1.005)*	1	1.193 (0.717–1.984)	1.240 (0.743–2.068)	1.710 (1.037–2.819)*

CI = confidence interval, LAP = lipid accumulation product, OR = odds ratio, PIR = poverty-income ratio.

* *P* < .05. Model 1: no covariates were adjusted. Model 2: age, race, and education level were adjusted. Model 3: age, race, marriage, education level, PIR, smoking, drinking, diabetes, and hypertension.

### 3.3. Smooth curve fitting and threshold effect between lap and endometriosis

**Figure 2.**
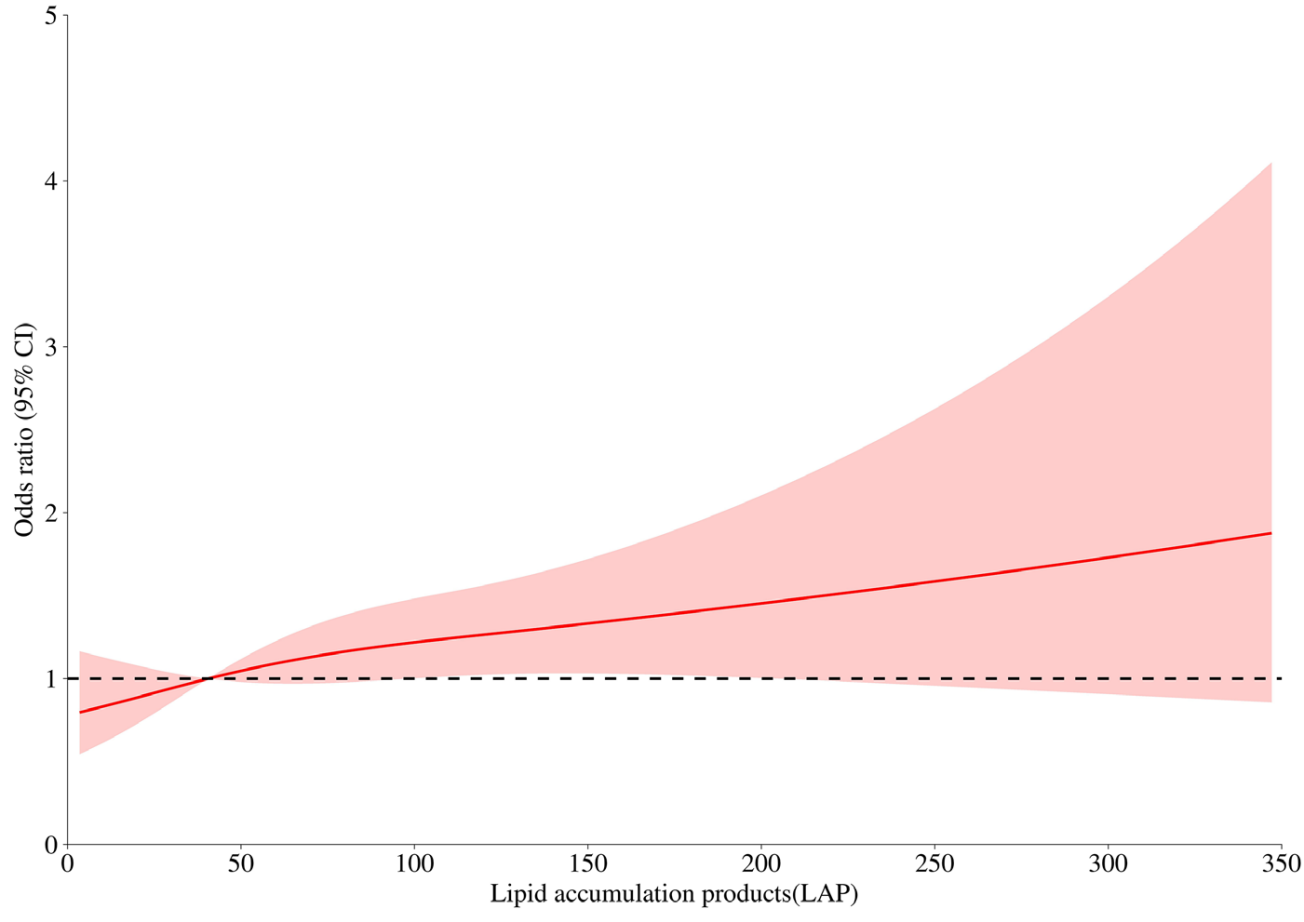
Dose–response relationship between LAP and endometriosis modeled using RCS. The smoothed curve illustrates the association between continuous LAP levels and disease risk, with shaded areas representing 95% CIs. CI = confidence interval, LAP = lipid accumulation product, RCS = restricted cubic spline.

RCS modeling identified a continuous and approximately linear dose–response pattern between LAP and endometriosis odds (Fig. 2, Table 3). In a linear model, higher LAP values were associated with higher odds of endometriosis (OR = 1.16, 95% CI = 1.03–1.23; *P* = .01). An exploratory segmented model with a data-driven knot at LAP = 44.60 yielded a similar positive slope above this value (OR = 1.13, 95% CI = 1.02–1.36; *P* < .001), but did not significantly improve model fit compared with the simple linear specification (likelihood ratio test *P* = .72), indicating no strong evidence for a true threshold effect. This trajectory supports a dose-dependent association between higher LAP values and greater odds of endometriosis in this cross-sectional sample.

**Table 3:** Analysis of threshold effect.

LAP	Adjusted OR (95% CI), *P* value
Model 1	
A straight-line effect	1.16 (1.03, 1.23), .01
Model 2	
Fold points (K)	44.60
LAP ≤ 44.60	1.21 (0.96, 1.53), .11
LAP > 44.60	1.13 (1.02, 1.36), <.001
Effect size difference of 2 versus 1	0.94 (0.67, 1.32), .72
Equation predicted values at break points	−2.50 (−2.59, −2.40)
Log likelihood ratio tests	0.72

CI = confidence interval, LAP = lipid accumulation product, OR = odds ratio.

### 3.4. Subgroup analysis

**Figure 3.**
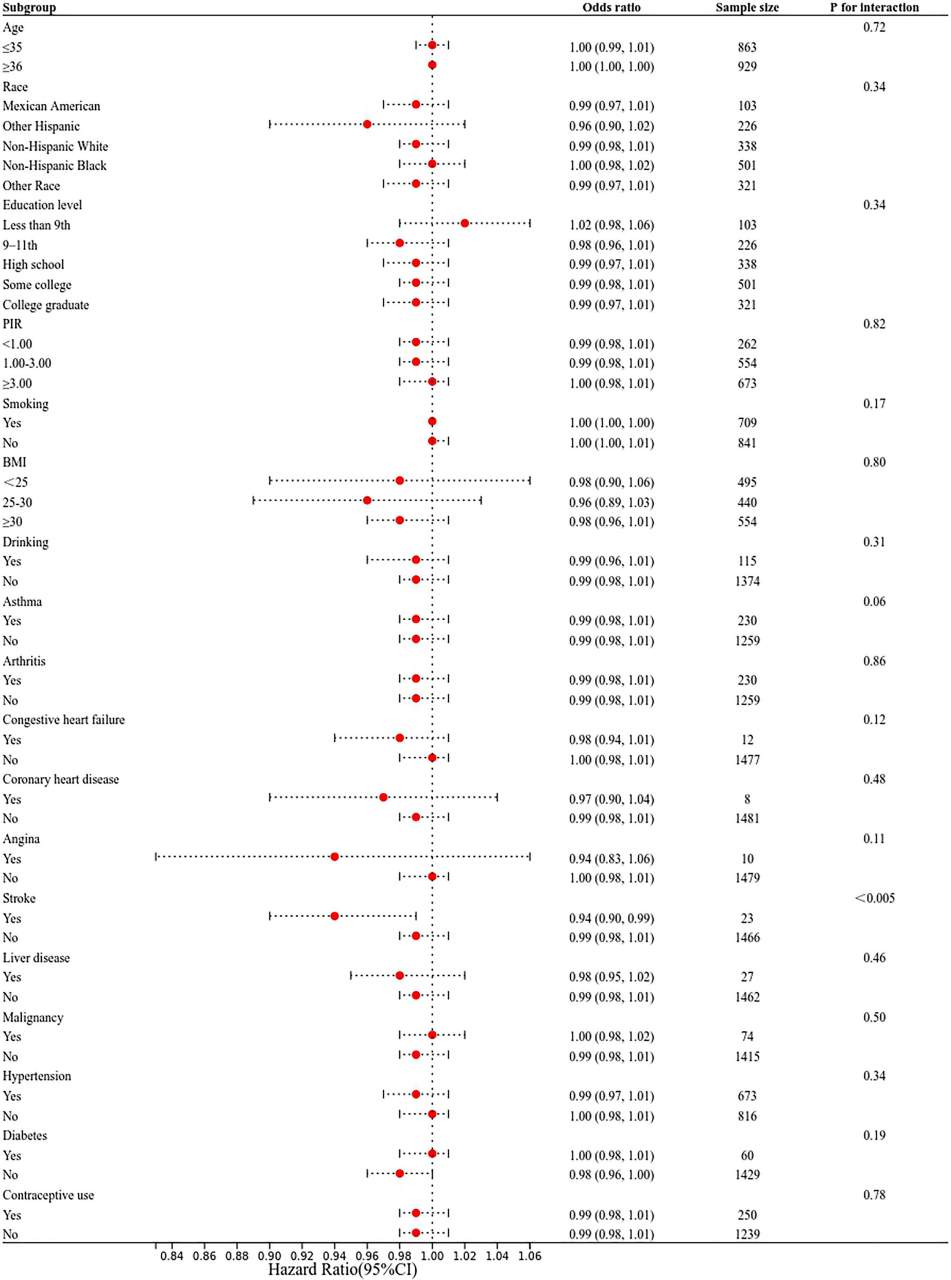
Forest plot presenting stratified analyses of LAP-endometriosis associations across subgroups. Multivariable-adjusted ORs with 95% CIs are shown for demographic and clinical subgroups. The vertical line at OR = 1 indicates no association. Most subgroups demonstrated consistent positive associations, with significant effect modification observed in the stroke history subgroup (interaction *P* < .05), suggesting an enhanced LAP-endometriosis association among stroke survivors. CIs crossing the null line indicate nonsignificant associations, while those entirely positioned to either side suggest significant risk-increasing or protective effects. BMI = body mass index, CI = confidence interval, LAP = lipid accumulation product, OR = odds ratio, PIR = poverty-income ratio.

To evaluate the stability of LAP-endometriosis associations across population strata, we performed prespecified subgroup analyses. Multiplicative interaction testing revealed homogeneous effects (*P* > .05) for age categories, racial/ethnic groups, educational attainment, smoking behavior, alcohol consumption, socioeconomic status, PIR, contraceptive methods, BMI, and comorbid conditions. However, stroke history displayed significant effect modification (*P* < .05), with strengthened LAP-endometriosis associations observed in post-stroke individuals compared with those without cerebrovascular events (Fig. 3).

## 4. Discussion

This nationwide cross-sectional study demonstrated a significant association between elevated LAP levels and the odds of endometriosis in a representative US cohort. Our analysis revealed 3 pivotal findings: a dose-dependent relationship where individuals in the highest LAP quartile faced 70% greater odds of endometriosis compared with the lowest quartile; an approximately linear dose–response pattern across the LAP distribution without strong evidence for a sharp threshold; and exploratory evidence of effect modification by stroke history, highlighting the complex interplay between metabolic dysregulation and neuroinflammatory pathways. These findings extend the emerging literature on the metabolic correlates of endometriosis and may have implications for risk stratification and clinical follow-up. Notably, because of the cross-sectional design and the absence of direct measurements of key biological mediators, mechanistic interpretations should be regarded as hypothesis-generating and grounded in prior literature rather than causal inferences from the present data.

One cross-sectional study based on the same database has previously reported an association between LAP and endometriosis.^[20]^ Compared with that study, the present analysis adopted a more refined analytical strategy by evaluating the association from both continuous and quantile-based perspectives, further incorporating RCS modeling and threshold effect analysis to characterize the dose–response pattern more systematically, and additionally exploring potential effect modifiers. Therefore, the present study is better interpreted as a supplementary and more detailed assessment built on existing evidence. The clinical significance of LAP as a metabolic biomarker compared to traditional indices like BMI requires integration with the diagnostic heterogeneity of endometriosis. Previous studies revealed that BMI-endometriosis associations differ by diagnostic method: elevated BMI showed no significant association with clinically confirmed cases,^[21]^ but overweight women (BMI ≥ 25 kg/m^2^) had a 29% increased risk of clinically suspected endometriosis. This discrepancy suggests BMI may reflect healthcare biases (e.g., conservative diagnosis of “suspected” cases in obese women) rather than direct biological mechanisms.^[22]^ In contrast, LAP integrates dual pathophysiological pathways – visceral adiposity (as measured by waist circumference) and lipid dysregulation (as indicated by triglycerides) – that have been implicated in endometriosis in prior studies. Visceral fat depots, unlike subcutaneous stores, have been reported to overexpress aromatase and 17β-hydroxysteroid dehydrogenase, potentially intensifying in situ conversion of adrenal androgens to estradiol and thereby contributing to higher local estrogen exposure.^[23]^ They simultaneously release pro-inflammatory adipokines (interleukin-6, tumor necrosis factor alpha, and leptin) that may skew macrophages toward the M1 phenotype.^[24]^ Hypertriglyceridemia may further exacerbate the microenvironment: TG-rich lipoproteins and free fatty acids undergo peroxidation, generating 4-hydroxynonenal and malondialdehyde, which can amplify reactive oxygen species and activate nuclear factor kappa B signaling in ectopic stromal cells.^[25]^ Taken together, these literature-based pathways suggest that higher waist circumference and TG burden could plausibly converge on estrogenic, inflammatory, and oxidative-stress signaling, which may help contextualize the observed dose-dependent association between LAP and endometriosis. However, these mediating processes (e.g., estradiol, adipokines, oxidative-stress markers, or nuclear factor kappa B activity) were not directly measured in NHANES, and thus the present study cannot determine whether such mechanisms account for the association. By reflecting metabolic abnormalities more accurately than BMI, LAP may offer clinicians a pragmatic marker for identifying women who could benefit from further metabolic evaluation when endometriosis is suspected.

The threshold effect analysis in this study provides additional insight into the association between LAP and endometriosis. Although initial observations suggested a potential nonlinear relationship at LAP = 44.60, rigorous statistical validation demonstrated the superior explanatory power of a simple linear model. Primary analyses revealed a consistent dose–response pattern, with higher LAP values associated with higher odds of endometriosis. This linear progression aligns with accumulating evidence that lipid metabolic disturbances exert gradational rather than threshold-dependent effects on gynecological pathologies,^[26,27]^ indicating that even subclinical metabolic dysregulation may incrementally contribute to disease progression.

While RCS curves identified an inflection point at LAP = 44.60, formal model comparisons favored the linear hypothesis. The segmented model showed no significant improvement over the linear model, and the effect difference across the inflection point was statistically nonsignificant. The absence of a definitive threshold does not diminish LAP’s clinical utility.^[28]^ The robustness of the linear model supports employing continuous LAP values for risk stratification, avoiding arbitrary dichotomization and reducing the likelihood of overlooking risk in individuals with moderate LAP elevations. Such a strategy parallels contemporary guidelines advocating continuous metabolic metrics for cardiovascular risk prediction,^[29]^ suggesting that analogous frameworks could optimize endometriosis prevention.

Subgroup analyses revealed stroke history as a possible effect modifier, with stronger LAP-endometriosis associations observed among women reporting prior stroke, although estimates were based on a small number of events and should be interpreted cautiously. Stroke survivors in the highest LAP quartile appeared to have higher odds of endometriosis compared with their non-stroke counterparts. This interaction may stem from post-stroke neuroendocrine dysregulation.^[30]^ Cerebral ischemia triggers hypothalamic–pituitary–adrenal axis hyperactivity, elevating glucocorticoids that exacerbate insulin resistance and lipolysis^[31]^ – processes that compound LAP’s metabolic toxicity. In addition, stroke-related immunosuppression may impair immune surveillance against ectopic endometrial implants.^[32,33]^ Animal models demonstrate that stroke-induced T-cell exhaustion permits unchecked growth of ectopic tissues, which suggests a plausible mechanism warranting investigation in endometriosis.^[34]^ Notably, stroke survivors exhibit altered gut microbiota composition, which modulates systemic inflammation and estrogen metabolism through enterohepatic recirculation.^[35,36]^ Although exploratory, these findings raise the possibility that a small subgroup of stroke survivors with elevated LAP could warrant closer gynecologic and metabolic follow-up.

Our findings intersect with emerging paradigms of endometriosis as a metabolic-inflammatory syndrome. Chronic low-grade inflammation, a hallmark of elevated LAP, creates a permissive microenvironment for ectopic lesion survival via multiple pathways. Pro-inflammatory cytokines stimulate angiogenesis and lesion vascularization,^[37]^ oxidative stress from lipid peroxidation damages peritoneal mesothelium, facilitating endometrial cell adhesion,^[38]^ and estrogen dominance driven by adipose aromatase activity promotes lesion proliferation while suppressing apoptosis.^[39]^ Therapeutic strategies targeting these axes, such as omega-3 fatty acids to reduce triglycerides or metformin to enhance insulin sensitivity, could disrupt this vicious cycle.^[40]^ A recent trial administering rosiglitazone reduced endometriosis-associated pain correlated with LAP decreases,^[41]^ supporting metabolic interventions’ potential. Furthermore, lifestyle modifications combining dietary changes with moderate exercise have shown efficacy in reducing both LAP and chronic pelvic pain severity in pilot studies.^[42]^

The clinical implications of our findings may lie primarily in risk awareness and metabolic profiling rather than in direct diagnostic application. LAP may be considered a readily available metabolic indicator in women undergoing evaluation for possible endometriosis, particularly when broader cardiometabolic risk assessment is also clinically relevant. However, whether LAP-guided monitoring or intervention improves endometriosis-related outcomes remains unknown and should be examined in prospective studies. A 6-month Mediterranean diet intervention reduced TG levels (0.09–0.15 mmol/L reduction) in older adults,^[43]^ while high-intensity interval training has been shown to improve insulin sensitivity and reduce visceral adiposity.^[44]^ Stroke survivors with elevated LAP may represent a higher-risk subgroup that warrants closer metabolic and gynecologic surveillance, although this signal is exploratory and based on few events. This potential interaction needs confirmation in larger, dedicated cohorts. Collaborative care models involving neurologists and gynecologists may support earlier recognition and more coordinated management in this population.

## 5. Strengths and limitations

Leveraging the NHANES database, this study provides additional nationwide evidence supporting an association between higher LAP and endometriosis, suggesting that LAP may be interpreted as a marker of metabolic risk rather than as a diagnostic criterion. NHANES’s standardized protocols and multidimensional measures strengthen internal validity, and its public availability bypasses many logistical hurdles of clinical datasets. Yet, the cross-sectional design precludes causal inference, leaving it uncertain whether LAP elevation precedes or follows disease onset. LAP should therefore be viewed as a risk-stratification tool that flags women needing further evaluation, not as a stand-alone diagnostic test. Longitudinal, surgically confirmed cohorts – ideally incorporating multi-omics and genetic data – are required to clarify temporality and to test whether adding LAP to routine assessment can shorten diagnostic delay. Caution is also warranted when generalizing these findings beyond the US population, and residual confounding cannot be excluded.

## 6. Conclusion

This study identifies a significant dose-dependent association between LAP and the odds of endometriosis, without strong evidence for a sharp threshold at any specific LAP value. LAP may be considered a potentially useful marker for metabolic risk stratification in women with possible endometriosis, but its clinical utility remains to be established in future prospective studies. Large-scale prospective studies and mechanistic investigations are needed to validate these findings and clarify the clinical relevance of this association.

## Acknowledgments

The authors thank the NHANES participants and staff for making the data publicly available. No individuals are named in this section.

## Author contributions

**Conceptualization:** Xin Liu, Qingbao Pang.

**Data curation:** Xin Liu, Qingbao Pang, Boyu Sun.

**Writing – original draft:** Xin Liu.

**Writing – review & editing:** Jing Lv.
